# Supplementary material for: A Near-Infrared Fluorescent Probe for Recognition of Hypochlorite Anions Based on Dicyanoisophorone Skeleton
Source: Molecules. 2023 Jan 3;28(1):402. doi: 10.3390/molecules28010402 (PMC9823594; doi:10.3390/molecules28010402)
Supplement: Supplementary file 1 [file molecules-28-00402-s001.zip › molecules-2092409-supplementary.pdf]

Supplementary Materials

A Near Infrared Fluorescent Probe for Recognition of Hypochlorite Anions Based on Dicyanoisophorone Skeleton

Chang-Xiang Liu [1,†], Shu-Yuan Xiao [1,†], Xiu-Lin Gong [1], Xi Zhu [2,*], Ya-Wen Wang [1,*] and Yu Peng [1]

[1]*School of Life Science and Engineering, Southwest Jiaotong University, Chengdu 610031, China*

[2]*Department of Neurology, the Third People's Hospital of Chengdu, the Affiliated Hospital of Southwest Jiaotong University, Chengdu 610031, China*

zhu61900@163.com; ywwang@swjtu.edu.cn

Table of Contents

1. General Methods. S2

2. Some reported work for the detection of ClO^-. S3

3. ^1H, ^{13}C NMR spectra and ESI-MS of **SWJT-9**. S6

4. Fluorescence intensity of **SWJT-9** in different solvents. S8

5. The effect of pH on fluorescence intensity. S9

6. The linear relationship between **SWJT-9** and different concentrations of ClO^-.... S10

7.Time-dependent experiment of **SWJT-9**. S11

8. ESI-MS spectrum of **SWJT-9** + ClO^-. S12

1. General Methods.

Isophorone, malononitrile, vanillin, Hexamethylenetetramine, trifluoroacetic acid, malonitrile were purchased from Beijing Innochem Company. All solvents were purchased from Tianjin Tianzheng Fine Chemical Reagent Factory Printing analysis. For fluorescence spectra, DMSO is analytically pure grade.

^{1}H and ^{13}C NMR spectra were measured with a Bruker avb-400 spectrometer, TMS was used as an internal reference, and ESI-MS spectra were measured with a Waters e2695 spectrometer. The fluorescence spectra were recorded by a Hitachi F-7000 spectrophotometer.

SWJT-9 (1.0 mM) stock solution was prepared in dimethyl sulfoxide. The test solution was prepared by placing 20.0 μL of the probe reserve solution in a four-way glass dish, then diluted to 2.0 mL with ethanol and then Ethanol /PBS buffer (9/1, V/V, pH=7.4) solution. Then add an appropriate amount of hypochlorous acid reserve solution. For all measurements, fluorescence spectra were excited at a wavelength of 550nm. It is worth noting that the preparation of peroxynitrite anion is shown in the previously reported literature [*].

HeLa cells were incubated in six-well plates for 24 h at 37 °C in an incubator. Cells were washed with phosphate buffered saline (PBS) and then 10.0 μM **SWJT-9** was added. Cells were incubated in a 37 °C incubator for 30 min, then washed with PBS and imaged. Subsequently 200.0 μM ClO^- was added and incubated in a 37 °C incubator for 30 min. HeLa cells were washed with PBS and imaged again. Fluorescence imaging of ClO^- within HeLa cells was recorded on a laser scanning confocal microscope. The excitation wavelength of the laser is 561 nm.

The prepared HeLa cell suspension was inoculated into a 96-well plate and cultured in a 5% CO_2 incubator at 37 °C for 24 h. Then the plate was washed twice with PBS buffer, 20.0 μL of **SWJT-9** at different concentrations was added to each well, and then the medium was added to make the concentrations of 0, 10.0, 20.0 and 50.0 μM in each well, respectively, and 6 duplicate wells were set at each concentration. After incubation in the incubator for 12 h, 20.0 μL of CCK-8 solution was added to each

well and the incubation was continued for 2 h. The absorbance at 450 nm was measured with a microplate reader to calculate the relative activity of each group.

[*] J.W. Reed, H.H. Ho, W.L. Jolly, Chemical synthesis with a quenched flow reactor Hydroxytrihydroborate and peroxynitrite, *J. Am. Chem. Soc.* **1974**, *96*, 1248-1249.

2. Some reported work for the detection of ClO^-.

Table S1. Compare our work with some reported work on ClO^- detection.

Probe		A_{max} (nm)	Ex/Em (nm)	Stokes shift	Response mode	Reference
**A**	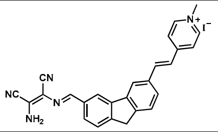	A_{max}=436	Ex=436<br>Em=541	105 nm	Turn-on	34
**B**	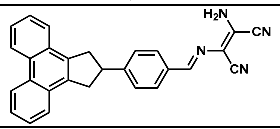	A_{max}=440	Ex=410<br>Em=500/625	185 nm	Ratiometric<br>500 nm on<br>625 nm off	35
**C**	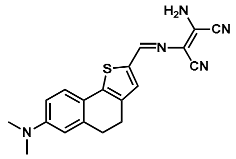	A_{max}=420	Ex=420<br>Em=500	80 nm	Turn-on	36
**D**	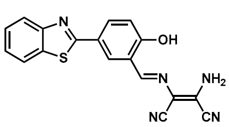	A_{max}=395	Ex=361<br>Em=480	85 nm	Turn-on	37
**E**	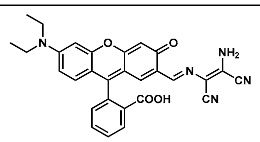	A_{max}=535	Ex=535<br>Em=577	42 nm	Turn-on	38
**F**	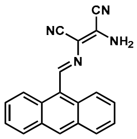	A_{max}=425	Ex=365<br>Em=432	7 nm	Turn-on	39
**G**	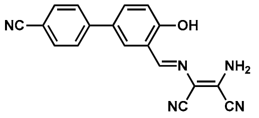	A_{max}=400	Ex=330<br>Em=500/607	207 nm	Ratiometric<br>500 nm on<br>607 nm off	40
**H**	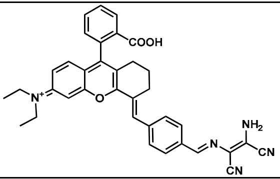	A_{max}=550	Ex=550<br>Em=667	117 nm	Turn-on	41
**I**	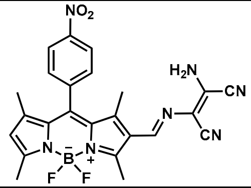	A_{max}=495	Ex=495<br>Em=525	30 nm	Turn-on	42
**J**	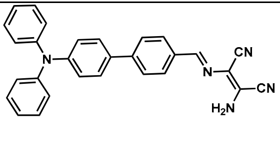	A_{max}=350	Ex=350<br>Em=440	90 nm	Turn-on	43

**K**	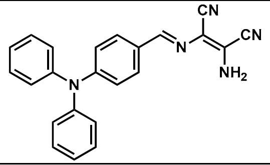	A_{max}=430	Ex=430<br>Em=587	157 nm	Turn-off	44
**L**	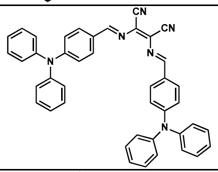	A_{max}=520	Ex=520<br>Em=615/677	157 nm	Ratiometric<br>615 nm on<br>675 nm off	44
**M**	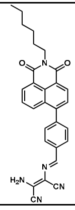	A_{max}=380	Ex=365<br>Em=440	60 nm	Turn-on	45
**N**	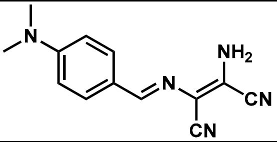	A_{max}=430	Ex=485<br>Em=521/635	91 nm	Ratiometric<br>521nm off<br>635nm on	46
**O**	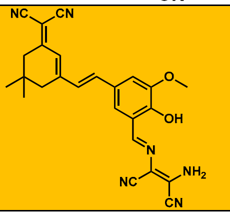	A_{max}=430	Ex=550<br>Em=667	237 nm	Turn-on	This work

3. ^{1}H, ^{13}C NMR spectra and ESI-MS of SWJT-9.

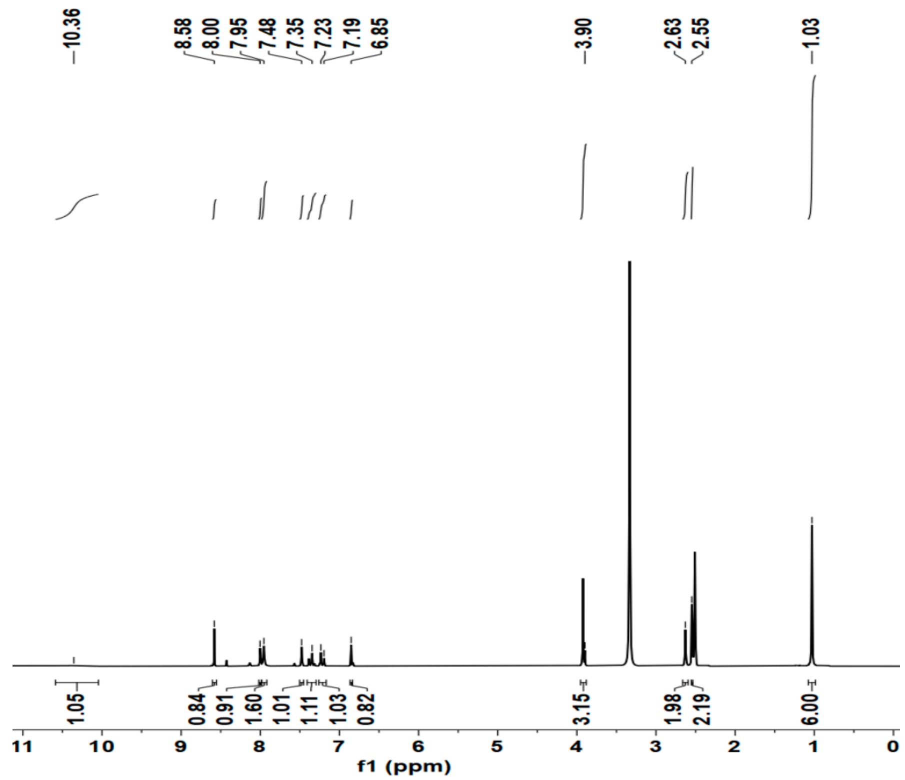

Figure S1. ^{1}H NMR spectrum (400 MHz, DMSO-d_6) of **SWJT-9**.

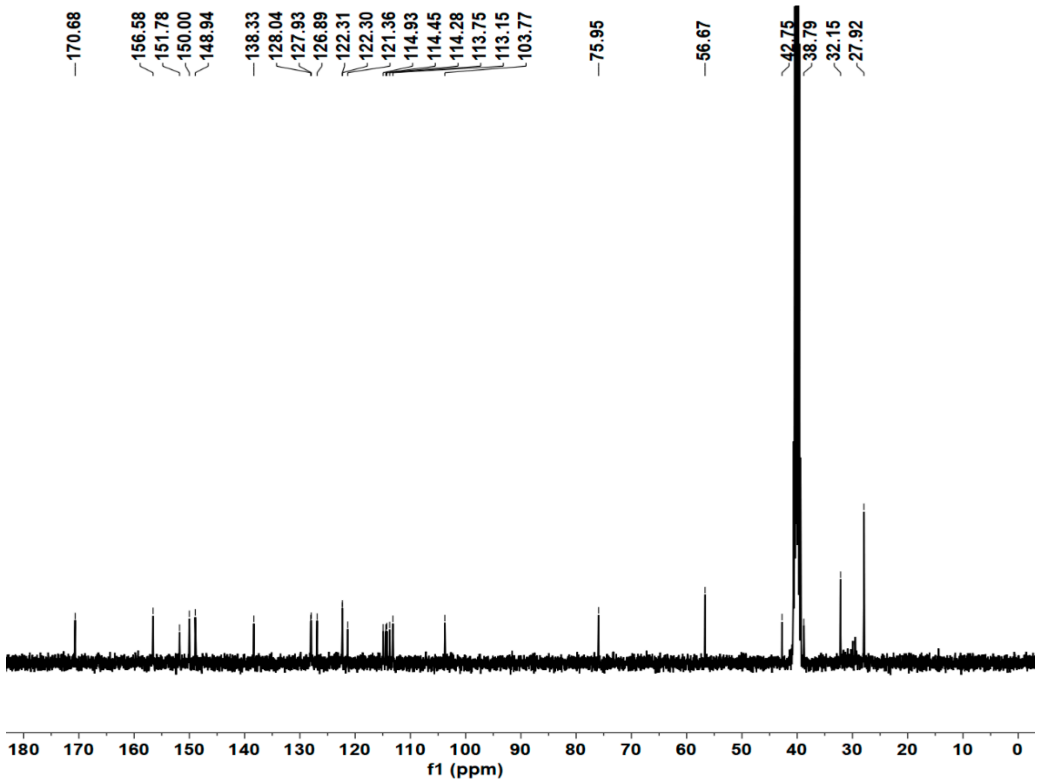

Figure S2. ^{13}C NMR spectrum (100 MHz, DMSO-d_6) of **SWJT-9.**

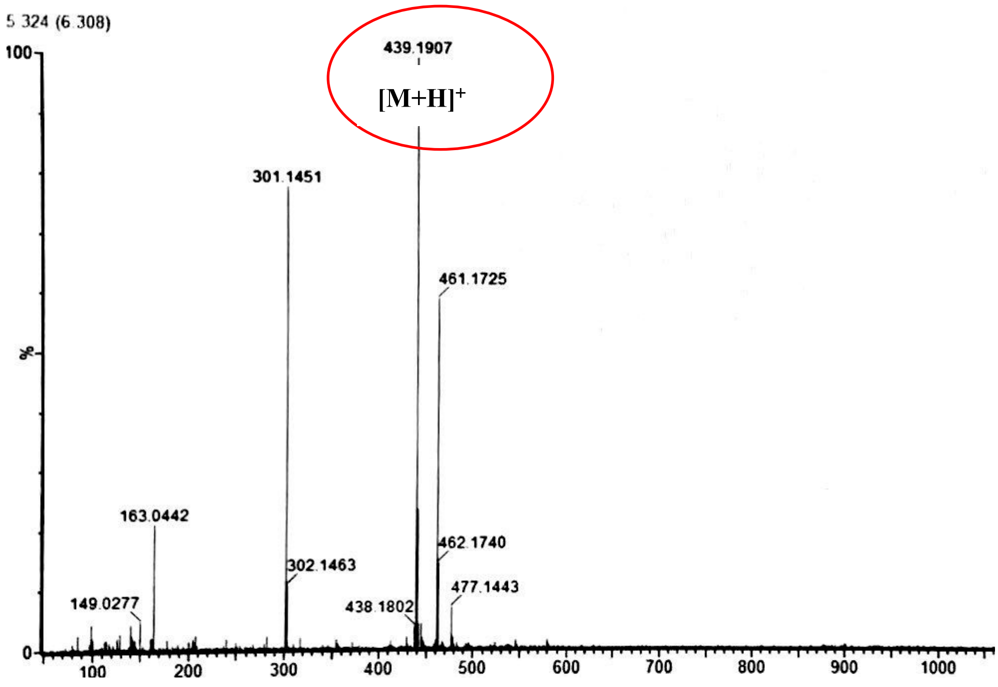

Figure S3. ESI-MS spectrum of **SWJT-9**.

4. Fluorescence intensity of SWJT-9 in different solvents.

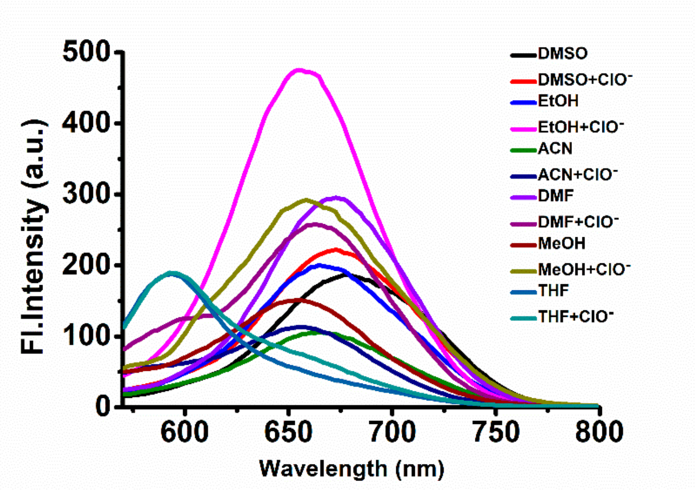

Figure S4. Fluorescence spectra of **SWJT-9** (10.0 μM) and **SWJT-9**+ ClO^- (2.0 mM) in dimethyl sulfoxide (DMSO), ethanol, acetonitrile (ACN), N,N-dimethylformamide (DMF) and tetrahydrofuran (THF).

5. The effect of pH on fluorescence intensity.

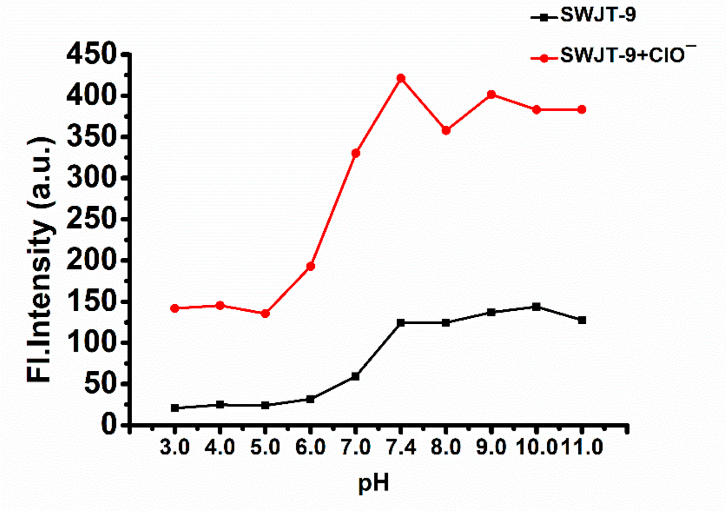

Figure S5. Fluorescence intensity of **SWJT-9** (10.0 μM) at 667 nm in the presence and absence of ClO^- (1.0 mM) in ethanol /PBS buffer (9/1, V/V) at different pH (3.0-11.0).

6. The linear relationship between SWJT-9 and different concentrations of ClO^-.

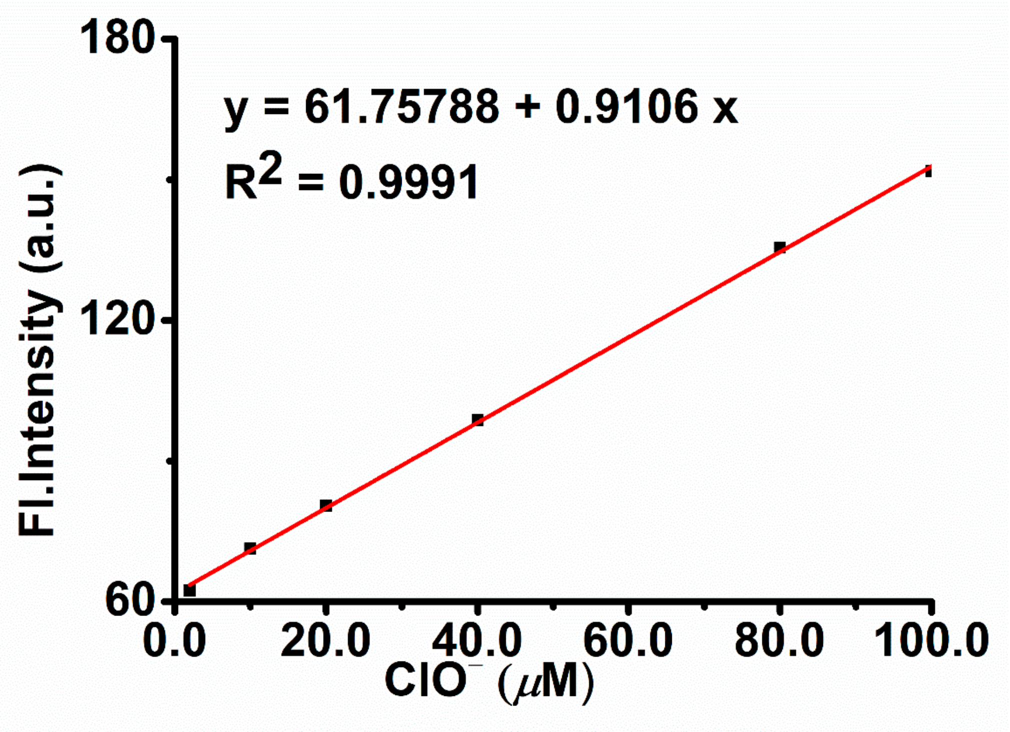

Figure S6. Linear relationship between fluorescence intensity of **SWJT-9** at 667 nm and concentration of ClO^-(0-100.0 μM).

The limit of detection (LOD) was calculated from the following equation based on fluorescence.

Titration results:

LOD = K ×δ/S

Where K = 2 or 3 (3 in this case), δ is the standard deviation of the blank solution, and S is the slope of the fitting line.

Linear Equation: Y=0.9106X + 61.75788

S = 0.9106 δ= 0.0075 K = 3

LOD = K ×δ/S = 0.0247 μm/L=24.7 nM

7.Time-dependent experiment of SWJT-9.

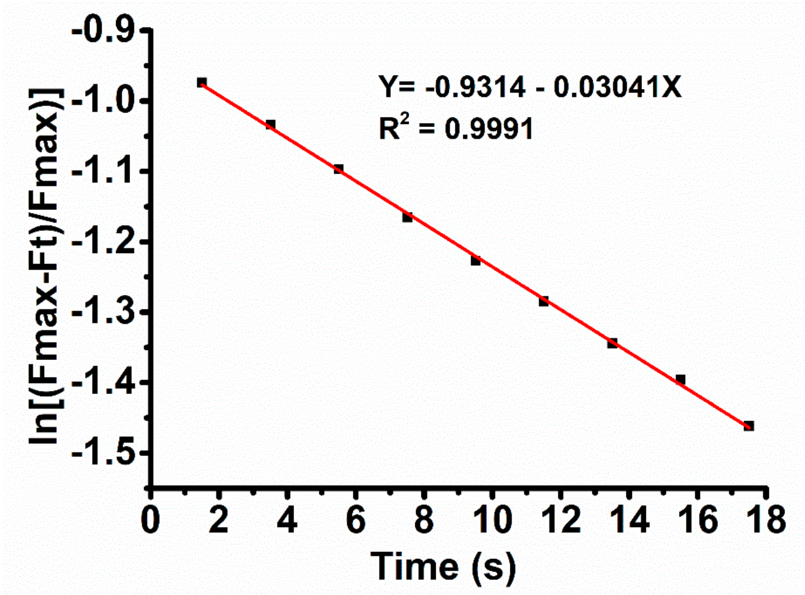

Figure S7. Quasi-first-order kinetic equations for **SWJT-9.**

The corresponding kinetic constants were determined using the following equation:

$\ln [(F_{max}-F_t)/F_{max}] = -k_{obs}\, t$

$t_{1/2} = \ln 2/k_{obs}$

Where F_{max} and F_t are the fluorescence intensity at maximum emission and time t.

$k_{obs} = 0.03041 s^{-1}$

8. **ESI-MS spectrum of SWJT-9 + ClO⁻.**

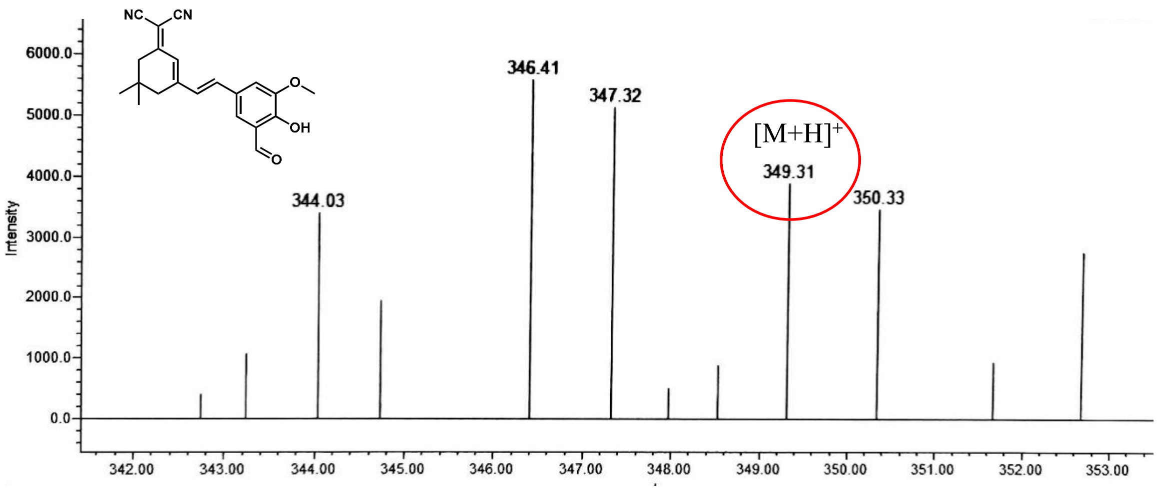

Figure S8. ESI-MS spectrum of **SWJT-9** + ClO⁻.
